# Ionic Liquid-Based Gels for Applications in Electrochemical Energy Storage and Conversion Devices: A Review of Recent Progress and Future Prospects

**DOI:** 10.3390/gels8010002

**Published:** 2021-12-21

**Authors:** Sharmin Sultana, Kumkum Ahmed, Prastika Krisma Jiwanti, Brasstira Yuva Wardhana, MD Nahin Islam Shiblee

**Affiliations:** 1 Department of Chemistry, Faculty of Science, Mawlana Bhashani Science and Technology University, Santosh, Tangail 1902, Bangladesh; sultana_chem@mbstu.ac.bd; 2 College of Engineering, Shibaura Institute of Technology, 3 Chome-7-5 Toyosu, Tokyo 135-8548, Japan; 3 Nanotechnology Engineering, Faculty of Advanced Technology and Multidiscipline, Universitas Airlangga, Surabaya 60115, Indonesia; prastika.krisma@stmm.unair.ac.id (P.K.J.); brasstira.yuva.wardhana-2020@stmm.unair.ac.id (B.Y.W.); 4 Department of Mechanical Systems Engineering, Yamagata University, 4 Chome-3-16 Jonan, Yonezawa 992-8510, Yamagata, Japan; nahin@yz.yamagata-u.ac.jp

**Keywords:** ionic liquid, ionic gel, battery, capacitor, fuel cell

## Abstract

Ionic liquids (ILs) are molten salts that are entirely composed of ions and have melting temperatures below 100 °C. When immobilized in polymeric matrices by sol–gel or chemical polymerization, they generate gels known as ion gels, ionogels, ionic gels, and so on, which may be used for a variety of electrochemical applications. One of the most significant research domains for IL-based gels is the energy industry, notably for energy storage and conversion devices, due to rising demand for clean, sustainable, and greener energy. Due to characteristics such as nonvolatility, high thermal stability, and strong ionic conductivity, IL-based gels appear to meet the stringent demands/criteria of these diverse application domains. This article focuses on the synthesis pathways of IL-based gel polymer electrolytes/organic gel electrolytes and their applications in batteries (Li-ion and beyond), fuel cells, and supercapacitors. Furthermore, the limitations and future possibilities of IL-based gels in the aforementioned application domains are discussed to support the speedy evolution of these materials in the appropriate applicable sectors.

## 1. Introduction

Continuous progress and comprehensive knowledge of the development of functional materials are essential to meet the demands of the rapidly growing energy sectors. Polymeric gels are a type of soft material that is gaining increasing attention in the electrochemical energy storage, conversion, sensing, gas sorption, and biomedical sectors [1,2,3,4,5,6]. Gels are three-dimensional networks of cross-linked polymeric units in liquid systems with variable pore sizes that exhibit exceptional structural derivability, including high water/solvent swellability, materials permeability, and tunable mechanical elasticity [7,8]. Additionally, a superior ability to serve as biocompatible materials and for transporting ions makes them potential candidates to be utilized in a wide variety of fields. Among various types of gels, IL-based gels, which incorporate ILs either as a solvent or as a polymerizable unit, have garnered substantial attention owing to their attractive properties derived from IL and their tunable functionalities. ILs are a unique type of liquid; they are mainly organic salts composed solely of organic cations and organic or inorganic anions with melting temperatures of less than 100 °C and have been well utilized in developing a variety of gels [9,10,11]. They are often classified as a green category of solvents (and electrolytes) and are distinguished by distinctive qualities, including negligible vapor pressure, high thermal stability, wide electrochemical potential windows, high ionic conductivity, and so on. Due to the tunable physical features of IL-based gels, they have broader prospects in energy applications. There is a growing need for clean and sustainable energy, particularly for energy storage and conversion materials and systems. Lithium batteries, supercapacitors, and fuel cells are typical examples, with their commercial applications including hybrid/electric/fuel cell automobiles, as well as being utilized in large-scale power supply systems such as multistoried building, hospitals, housing apartments that are already underway. Extensive and ongoing research has contributed to the development of novel materials employed in these devices. Furthermore, IL-based materials are popular not only in Li-ion batteries, but also in Li–sulfur and Li–oxygen batteries, owing to being less prone to side reactions, degradation, and evaporation (solvent) compared to traditional carbonate-based electrolytes. Water-assisted proton conductivity of proton conducting membranes, primarily Nafion, impedes fuel cell use at temperatures above 100 °C due to the rapid evaporation of water and associated reduction in proton conductivity. These problems have sparked research into new electrolyte materials based on ILs. Meanwhile, because of their nonvolatility and high thermal stability, ILs have been employed as carbon material precursors in some cases. This innovative method uncovered functional and designable carbon molecules for energy applications [12].

This manuscript compiles and examines the most recent scientific advances in the synthesis and application of IL-based gels. While IL liquid-based gels have a wide range of applications in energy storage and conversion, sensors, actuators, wearable devices, gas absorption, and biomedicine, this article will mainly focus on the latest developments and applications of IL-based gels in the energy storage and conversion sectors, and their future prospects will be discussed to guide researchers in choosing the most effective strategies for developing and enhancing research in pertinent domains. 

## 2. Preparation of IL-Based Gel

### 2.1. Classification of Preparation Methods

The classification of IL-based gels or ionogels and the different routes to synthesize IL-based gel electrolytes or ionogels have been reviewed by a number of research groups [13,14,15,16]. The various kinds of IL-based gels can be simply categorized as physical gels and chemical gels according to the type of matrix formation [13,14,15]. In physical gels, internal, three-dimensional, cross-linking networks occur due to weak reversible interactions such as hydrogen bonds, hydrophobic interactions, van der Waals interactions, π-π interactions, crystallite junctions, etc., whereas in chemical gels the cross-links are formed by covalent bonding. Typically, chemical gels offer good mechanical strength compared to physical gels. Another type of IL-based gel classification is organic, inorganic, and hybrid organic–inorganic IL-based gels [16]. Until now, various gel techniques to confine and immobilize ILs into a three dimensional organic or inorganic matrix have been reported, such as the sol–gel process, polymerization, photopolymerization, and solvent casting [16,17,18]. Among them, the sol–gel process has gained much interest for producing IL-based gel electrolytes with enhanced mechanical properties for numerous electronic applications related to lithium-ion batteries, supercapacitors, and other electrochemical devices [15,18,19,20]. Moreover, the sol–gel derived IL-based gels have been reported to be used in electrochromic, drug-delivery, photo-stimuli, and luminescence systems in recent years [21,22]. Meanwhile, polymerization leading to the formation of micro-patternable gel electrolytes, has received significant attention due to its promising application in microdevices such as microcapacitors, flexible sensors, and 3D printing technology [18,23]. In the following section, several ways for obtaining IL-based electrolytes are presented.

#### 2.1.1. Sol–Gel Method

A wide variety of IL-based gels, including chemical gels and physical gels, has been successfully synthesized via the sol–gel process to date [24,25,26]. The sol–gel process is a simple and low-toxic synthesis technique that generally involves hydrolysis and polycondensation of metal alkoxide precursor in IL [24]. Typically, the sol–gel process can be divided into stages as sol formation, gelation, aging, and drying. The resulting material produced by sol–gel reaction in IL, termed as ionogel or IL-based gel, comprises a continuous mesoporous matrix with an interstitial liquid phase, the IL, that fills the pores. While drying the gel under ambient conditions, the volatile species produced from hydrolysis and condensation is removed.

**Figure 1:**
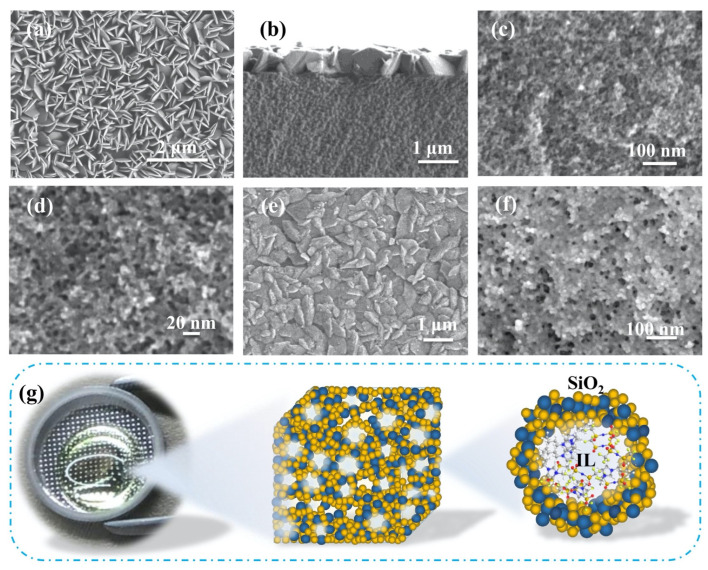
(**a**) FESEM image of the surface of IE-T electrolyte; (**b**) FESEM image of the cross-section of IE-T electrolyte; (**c**,**d**) FESEM images of the IE-T electrolyte sample after ionic liquid has been extracted; (**e**) FESEM image of the surface of IE-B electrolyte; (**f**) FESEM image of the IE-B sample after ionic liquid has been extracted; (**g**) photograph of IE-T electrolyte and schematic of internal microscopic three-dimensional structure of ionogel. Reprinted with permission from ref. [30].

In 2017, Ashby et al. prepared an IL-based gel by the sol–gel process from 1-butyl-3-methylimidazolium bis(trifluoromethanesulfonyl)imide (BMIMTFSI) IL containing LiTFSI and silica (SiO_2_) sources as a mixture of tetramethoxysilane (TMOS) and triethylvinylorthosilicate (VTEOS) with formic acid catalyst in cyclohexane [20]. The ionic conductivity of the prepared IL-based gel was reported to be 2 mS cm^−1^, which was comparable to that of neat IL (3 mS cm^−1^). Later in 2019, they developed a LiOH-modified IL-based gel employing a two-step sol–gel process with TMOS, VTEOS, and formic acid as SiO_2_ precursor solution and 0.5 M LiTFSI in BMIMTFSI IL at –20 °C [27]. This study showed that the LiOH-modified IL-based gel has slightly lower ionic conductivity than that of original IL-based gel due to a decreased surface area and size of the pore of modified gel with the attendance of LiOH. In 2018, Li’s group reported a one-step sol–gel method to synthesize an organosilicon-functionalized IL-based gel by in situ immobilization of a TFSI-based IL composed of 1-trimethylsiylmethyl-3-butylimidazolium (SiMC_4_Im) containing 0.6 mol kg^−1^ LiTFSI within a nanoporous SiO_2_ matrix [28]. The as-prepared IL-based gel exhibited a wide electrochemical potential window of 4.87 V (vs. Li/Li) at room temperature and a conductivity of 0.18 mS cm^−1^. The IL-based gel showed significantly lower ionic conductivity than the pristine IL. The trends in the values of IL-based gels and neat ionic IL observed in this study were similar to those of previously reported data [20,27]. Meanwhile, Asbani et al. developed a thick, self-standing IL-based gel from a sol made of TMOS, dimethyl-dimethoxysilane (DMDMS), formic acid, and the IL 1-ethyl-3-methylimidazolium bis(trifluoromethanesulfonyl)imide (EMIMTFSI) where the [TMOS/DMDMS]/(EMIMTFSI)–formic acid molar ratio was [70/30]/50:1 at room temperature [29]. Recently, Cheng and coworkers prepared a flower-like structured IL-based gel using lithium trifluoromethanesulfonate (LiOTf) or LiTFSI salt mixed with tetraethylorthosilicate (TEOS) in 1-butyl-3-methylimidazolium tetrafluoroborate (BMIMBF_4_) IL with dilute hydrochloric acid solution as a catalyst [30]. The two gels obtained from LiOTf and LiTFSI salt were named as IE-T and IE-B, respectively. Field emission scanning electron microscopy (FESEM) images of gel electrolytes are displayed in Figure 1a–d (IE-T) and Figure 1e–f (IE-B). The IE-T exhibits a regular and flower-like sheet structure, whereas, for IE-B, the flower-like sheets were not regular and complete in comparison with IE-T. A schematic representation of the three dimensional structure of synthesized IL-based gel electrolyte is shown in Figure 1g. From the electrochemical measurements, the conductivity of IL-based gels with LiOTf and LiTFSI salts was estimated to be 1.21 mS cm^−1^ and 1.19 mS cm^−1^, respectively, at 30 °C. Earlier, a research work by Chen et al. demonstrated a one-step sol–gel method to design a biometric ant-nest IL-based gel electrolyte from N-propyl-N-methylpyrrolidinium bis(trifluoromethylsulfonyl)imide (Pyr_13_TFSI) containing LiTFSI and 3- methacryloxypropyltrimethoxysilane in formic acid [31]. The IL was immobilized within a biomimetic ant-nest SiO_2_ matrix (X-SiO_2_) by in situ sol–gel of the silane coupling agent. The IL-based gel with 2 M LiTFSI displayed higher ionic conductivity compared to that of the neat IL electrolyte. By contrast, the SiO_2_/IL electrolyte showed much lower ionic conductivity than that of the IL electrolyte. By adapting the acid-catalyzed sol–gel method, Chen’s research group successfully prepared a silica-based nanocomposite IL-based gel electrolyte from TEOS precursor and 1-butyl-1-methylpyrrolidinium bis(trifluoromethylsulfonyl)imide (BMPTFSI) IL containing LiTFSI in water and 1-methoxy-2-propanol mixed solvent [32]. This study showed that with the increase in volume fraction (*x*) of IL electrolyte in the nanocomposite gels, the ionic conductivity exceeded that of the pure IL itself by more than 200% for the highest *x* values (*x* = 2.0). Beside SiO_2_-based IL-based gel, in 2016, Wu and his team reported the non-aqueous sol–gel synthesis of IL-based gel offering ionic conductivity of about 1 mS cm^−1^ based on a titanium alkoxide precursor, tetrabutyl titanate (TBOT), and solubilized in EMIMTFSI IL electrolyte [25]. The sol–gel procedure is beneficial for creating IL-based gels because it allows for the use of both the variety of the sol–gel process and IL chemistry. The sol–gel approach provides access to a broad range of supports, from pure metal oxides to organic–inorganic silica-derived hybrids, as well as the unique benefit of ILs with adjustable characteristics for particular requirements.

#### 2.1.2. Polymerization Method

There have been a number of reviews on the preparation of IL-based gels by in situ polymerization or cross-linking of monomers dissolved in IL [13,14,16,17,18]. Generally, the in situ polymerization process involves dissolving a polymerizable monomer in ILs with or without the use of a cosolvent and followed by the removal of the cosolvent. Free radical, chain, step, thermal, and photopolymerization are the common techniques employed to synthesize IL-based polymeric gels [13,14,23]. Another route is the soaking method, which involves the swelling of a polymer in IL or by preparing polymer film in multistep procedures and then soaking the as-prepared film with IL [33]. The IL-based polymer–IL-based gels can also be obtained via the solution casting method, which typically includes the dissolution of polymer and IL in a volatile solvent followed by the casting and drying of the homogenous mixture to remove the solvent. Yang et al. [34] summarized the design and preparation of polymer/inorganic hybrid electrode (HE), as well as the enhancement of its electrochemical characteristics.

Recently, Zhang et al. [35] reported the preparation of robust IL gel electrolytes for a supercapacitor by dissolving Poly(vinylidene fluoride-co-hexafluoropropylene) polymer (P(VDF-HFP)) in two promising ILs, 1-ethyl-3-methylimidazolium bis(trifluoromethylsulfonyl)imide (EMIMTFSI) and 1-butyl-1-methylpyrrolidinium tris(pentafluoroethyl)trifluorophosphate [C_4_mpyr][eFAP], respectively, with stirring at 70 °C for 12 h and drying at 50 °C. Finally, the samples were stirred vigorously for 0.5 h above their sol–gel temperature and followed by cooling again to 50ºC. The minimum concentration of P(VDF-HFP) polymer needed to form an IL-based gel was reported to be 4 wt% for these, EMIMTFSI and [C_4_mpyr][eFAP]. The ionic conductivities of EMIMTFSI-based and [C_4_mpyr][eFAP]-based IL-based gels were estimated to be 8.6 and 1.0 mS cm^−1^ at 25 °C, respectively, with a very minor loss of conductivities compared to those of pure ILs, EMIMTFSI (9.5 mS cm^−1^) and [C_4_mpyr][eFAP] (1.2 mS cm^−1^). In 2019, Zhong and coworkers [23] prepared a 3D IL-based gel by mixing thiol, acrylate monomers, trimethylolpropane tris(3-mercoptopropianate), and polyethylene glycol diacrylate (PEGDA) in triethylamine (TEA) catalyst in the presence of EMIMTFSI using the Michael addition polymerization process. The resulting IL-based gel with 50% wt of IL showed remarkable ionic conductivity in the range of 0.1 mS cm^−1^, and the prepared IL-based gels were suggested as advantageous for fabricating soft robotics, soft actuators, flexible and stretchable electronics, and medical devices. Recently, in 2021, Malunavar’s group designed an IL-based gel electrolyte for sodium battery applications by mixing N-ethyl-N-methylpyrrolidinium bis(fluorosulfonyl)imide (C_2_mpyrFSI) IL with NaFSI supported on a mat of electrospun poly (vinylidene fluoride) (PVDF) nanofibers [36]. Meanwhile, Wang and his team developed a novel trimethylolpropane trimethylacrylate (TMPTMA)-based IL-based gel electrolyte via in situ thermal polymerization using lithium bis(trifluoromethane)sulfonimide (LiTFSI), dimethylcarbonate (DMC), and IL (IL)-tributylmethylammonium bis(trifluoromethanesulfonyl) imide (N_1,4,4,4_ TFSI) [37]. The TMPTMA monomer and initiator (2,2-Azobis-(2,4-dimethylvaleronitrile)) (ABVN) were dissolved into the mixture of plasticizer, N_1,4,4,4_ TFSI, LiTFSI, and DMC by ultrasonic stirring for 30 min to obtain the precursor solution. The as-formed precursors were polymerized at 80 °C for 30 min to obtain transparent IL-based gel electrolytes for lithium-ion battery applications.

**Figure 2:**
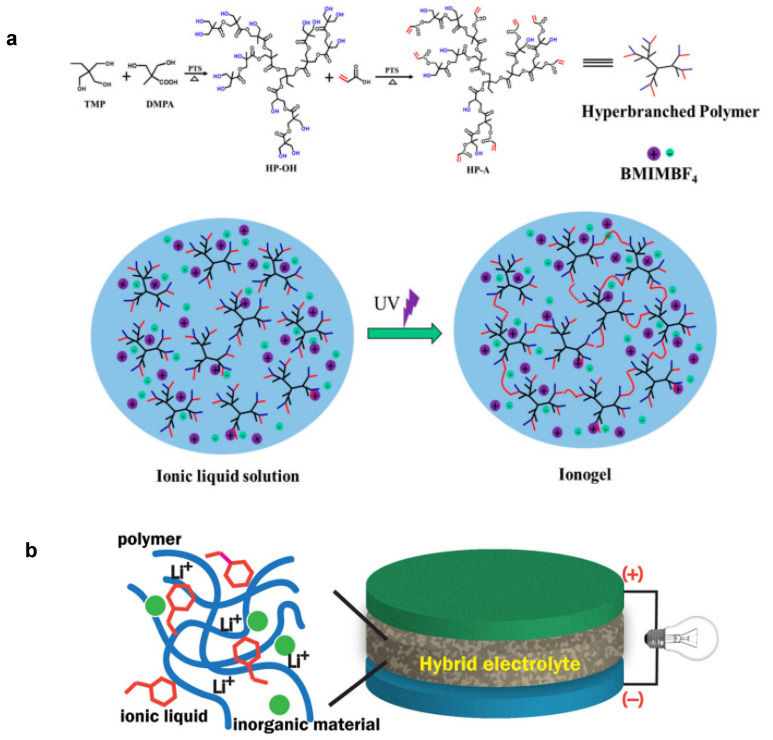
(**a**) Synthesis HP-A and IL-based gel from HP-A by photopolymerization [44] and (**b**) basic overview of polymer/inorganic hybrid electrolyte (HE) [34].

Photopolymerization has been used extensively for fabricating IL-based gel electrolytes in recent years. This process offers a facile and quick synthesis route. A study published in 2018 reported the preparation of a flexible and highly conductive IL-based gel by in situ UV-initiated free-radical polymerization of thiolacrylate precursors in the presence of EMIMTFSI of various proportions [38]. Another work by Cao and colleagues [39] showed the fabrication of a hydrophobic, robust, and ultrahighly stretchable IL-based gel using an ex situ method that was the photopolymerization of the monomer and subsequent swelling of the polymer in IL. For fabrication of the IL-based gel ethyl acrylate (EA), ethylene glycol dimethacrylate (EGDMA) and phenylbis(2,4,6-trimethylbenzoyl)phosphine oxide (PBPO) as the monomer, cross-linker, and photoinitiator, respectively, were used. Firstly, the photopolymerization of EA monomer solution in EGDMA and PBPO was conducted to obtain a poly(ethyl acrylate) PEA elastomer. Finally, EMIMTFSI IL was incorporated into the elastomers to obtain a transparent IL-based gel. The as-prepared IL-based gel exhibited outstanding durability while utilized in a skin-like sensor under harsh environmental conditions. Meanwhile, Biria’s team described the preparation of a highly conductive IL-based gel electrolyte via photopolymerization using a mixture of PEGDA polymer and camphorquinone and (4-octyloxyphenyl)phenyliodonium hexafluoroantimonate) as the photoinitiator in 1-ethyl-3-methylimidazolium tetrafluoroborate (EMIMBF_4_) containing calcium salt as solvent [40]. Recently, in 2019, Rana et al. synthesized an ultradurable double network (DN) IL-based gel composed of densely cross-linked polyvinyl alcohol (C-PVA) as the first network and coarsely cross-linked poly(2-hydroxyethyl methacrylate) (C-pHEMA) as the second network in EMIMBF_4_ as solvent and K_2_S_2_O_8_ as initiators via a two-step, thermally-initiated, free-radical polymerization [41]. Then, in 2020, Sun and coworkers developed a physically linked DN IL-based gel for a flexible bimodal sensor via interpenetrating a poly- (hydroxyethyl acrylate) (PHEA) into an agarose network in 1-ethyl-3-methylimidazolium chloride (EMIMCl) [42]. The mixture of hydroxyethyl acrylate (HEA) and EMIMCl were heated and magnetically stirred for 5 min to obtain a homogeneous solution. Then, agarose was added to the solution and stirred at 85 °C and cooled to room temperature to obtain an agarose IL-based gel. The DN IL-based gel was finally produced by the photopolymerization of HEA monomer after irradiating under a 365 nm LED light intensity of 80 mw/cm^2^ for 5 min. Similarly, by using in situ photopolymerization, Ming’s group reported the synthesis of a transparent, stretchable, phase-transformable 1,3-dimethyl imidazolium bis(trifluorosufonylmethane imide) (MMITFSI)-based IL-based gel for long-storage capacitors from a precursor solution consisting of butyl acrylate and fluorinated butyl acrylate as monomers, 1-hydroxycyclohexyl phenyl ketone (Irgacure 184) as the photoinitiator, and 1,6-hexandiol diacrylate (HDDA) as the cross-linker [43]. Meanwhile, Hyun et al. illustrated the development of layered heterostructure IL-based gel electrolytes based on EMIMTFSI and 1-ethyl-3-methylimidazolium bis(fluorosulfonyl)imide (EMIMFSI) containing 1 M LiTFSI salt using hexagonal boron nitride (hBN) nanoplatelets as the gelling matrix [44]. The designed layered heterostructure IL-based gels hold high ionic conductivity of >1 mS cm^−1^ at room temperature. Zhao et al. conducted photopolymerization to produce an IL-based gel based on hyperbranched polymer aliphatic polyester with acrylate terminal groups in BMIMBF_4_ with superior ionic conductivity for the fabrication of gel polymer electrolytes for applications in lithium-ion batteries [45]. The synthesis of this IL-based gel was carried out in two stages, which are illustrated in Figure 2a. HP-OH was obtained through a quasi-one-step reaction of 2, 2-bis(hydroxymethyl)propionic acid (DMPA), 2-ethyl-2-(hydroxymethyl)-1,3-propanediol (TMP), and a number of catalytic PTS, and then synthesized to create acrylic terminated hyperbranched polymer (HP-A). To make the IL-based gel electrolyte, 30% HP-A is photopolymerized with the photoinitiator, 10% LiBF4 salt, 50% IL BMIMBF4, and 10% PVDF is dissolved in N,N-dimethylformamide (DMF). On the other hand, Muench and coworkers adopted a UV-induced polymerization to form IL-based gel film comprised of benzyl methacrylate (Bn-MA) and poly(ethylene glycol) methylether methacrylate (mPEG-MA) with the cross-linker triethylene glycol dimethacrylate (TEG-DMA) in BMIMTFSI [46]. By employing UV-curing, Porthault et al. prepared an IL-based gel from bisphenol A ethoxylate dimethacrylate (BEMA) and poly(ethylene glycol) methyl ether methacrylate (me-PEGMA) in LiTFSI/N-propyl-N-methylpyrrolidinium bis(trifluoromethanesulfonyl) imide (Pyr_13_TFSI) IL electrolyte. From the experimental results, the ionic conductivity of the IL-based gel was reported to inversely decrease with salt content from 4.12 to 0.25 mS cm^−1^ for the molar ratio of Pyr_13_TFSI: *x* LiTFSI of 0.0 and 0.6, respectively, at 25 °C [47].

Previous publications have also revealed that the immobilization of IL into a polymer network can be achieved through solution casting method [48,49,50,51,52]. Chen et al. drop casted a mixture of acetone and EMIMTFSI IL into P(VDF-HFP) copolymer to produce a thin film, which was further soaked into 1.0 LiTFSI solution in a mixed solvent of dimethoxyethane/dioxolane (DME/DOL) to obtain the IL-based gel electrolyte. The ionic conductivity of the prepared IL-based gel was 1.5, 2.6, 3.6, 4.6, and 5.5 mS cm^−1^ at 35, 45, 55, 65, and 75 °C, respectively; these were higher compared to those of LiTFSI-P(VDF-HFP) without immobilized EMIMTFSI [48]. Yang’s team developed a polymer IL-based gel consisting of P(VDF-HFP) in N-methyl-pyrrolidone (NMP) solvent with the sequential addition of Pyr_13_TFSI IL containing LiTFSI salt [49]. The obtained solution was then drop casted into a clean glass plate to form a film. The authors have further tested the IL-based gel performance as an electrolyte in lithium ion battery. Ravi et al. designed another gel polymer electrolyte comprised of P(VDF-HFP)/LiTFSI/Pyr_13_TFSI using a standard solution casting technique [50,51,52]. The authors concluded that by increasing the LiTFSI and Pyr_13_TFSI content, the ionic conductivity of the IL-based gel increases and reaches a maximum value of 0.693 mS cm^−1^ at 25 °C when the IL-based gel contains 30 wt% LiTFSI and 60 wt% Pyr_13_TFSI. Recently, in 2020, Muthupradeepa employed the solution casting method to synthesize a polymer gel electrolyte with P(VDF-HFP) and TFSI-based IL containing trihexyltetradecylphosphonium (P_14,6,6,6_) cation at different ratios of polymer to IL [53]. Similarly, Yao et al. fabricated an IL-based gel electrolyte from P(VDF-HFP) and LiTFSI/EMIMTFSI via a simple solution casting method [54]. To fabricate the IL-based gel electrolyte, the P(VDF-HFP), EMIMTFSI IL, and SiO_2_ were dissolved in acetone by stirring at 40º C for 3h. The LiTFSI was added to the solution and stirred for 10 min. Finally, the obtained solution was casted on to a polytetrafluoroethylene mold and dried at 70 °C overnight and the IL-based gel electrolyte was stored in a glovebox. Recently, Xing and colleagues demonstrated the preparation of IL-based gel electrolyte consisting of P(VDF-HFP) dissolved in acetone, EMIMBF_4_ IL, and bis(trifluoromethane) sulfonimide sodium (NaTFSI) salt with different dosages of IL [55]. A significant improvement in the conductivity of the IL-based gel electrolyte was realized by increasing the IL content in the electrolyte. These findings were in good agreement with the experimental results described in previous reports [47,53]. Meanwhile, by utilizing a simple solution casting method, Fan and colleagues developed a redox-active IL-based gel composed of 1-butyl-3-methylimidazolium iodide (BMIMI) IL, PVDF-HFP, and carbon nanotubes (CNTs) for application in supercapacitors [56].

IL-based gels, which are composed of polymeric gelators and ILs, are another potential platform that can offer many possible approaches, with vast research scopes. The properties of the co-polymer gelators are a critical parameter that is directly related to gel performance [57,58,59,60,61]. Gelation should be accomplished with the lowest quantity of polymer possible to generate highly conductive gels. Thus, coherent molecular design is required to include both IL-soluble and IL-insoluble components [59,60,61]. When combined with IL, the IL-soluble segments swell, forming ion conductive channels, while the components that are insoluble in ILs aggregate to limit enthalpically unfavorable interaction with the ILs. Various copolymers such as block [62,63,64,65], star [57,58], and random [59,60,61] types of ion gel have been proposed based on these requirements.

**Figure 3:**
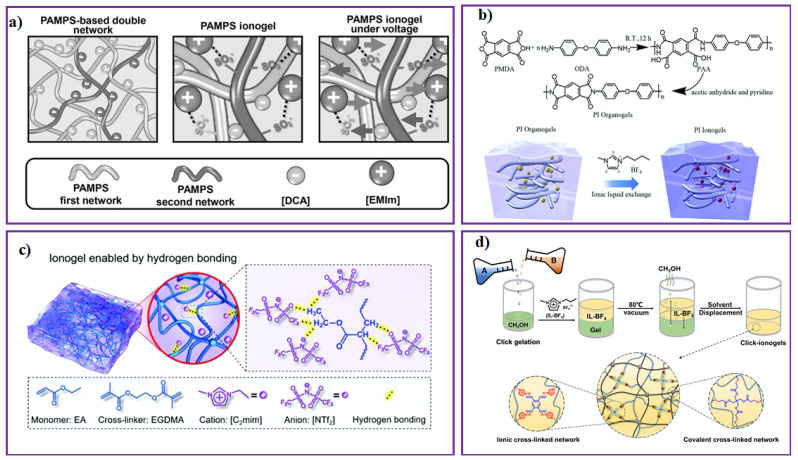
(**a**) Locking 1-ethyl-3-methylimidazolium dicyanamide (EMIMDCA) IL in poly(2-acrylamido-2-methyl-1-propanesulfonic acid) (PAMPS)-based DN network through electrostatic interactions [70,71]. (**b**) Forming polyimide ionogels by solution displacement attributed to hydrogen bonding [72]. (**c**) Locking EMIMTFSI IL into the PEA elastomer network through hydrogen bonding [39]. (**d**) The thiol–ene click reaction for preparing DN click ionogel [68].

Other methods for IL-based gel preparation, namely solvent exchange, click reaction, electrostatic interactions, electro-spinning, 3D printing, H-bonding, mechanical ball milling, and roller pressing, are available in the literature [15,66,67,68,69,70,71,72]. Figure 3 shows different methods of obtaining IL-based gels. There are still some challenges in the development of IL-based gels due to limitations of the network, such as fewer free ions in IL-based gels, which leads to lower conductivity of the gel electrolytes compared to pure ILs. In addition, a humid environment causes an unstable electrical performance of the IL-based gels containing hydrophilic ILs and polymer chains. However, with the recent development of technology, a large number of IL-based gels have been successfully developed by various preparation methods for application in energy storage and flexible wearable electronics.

Although each method offers various advantages such as simplicity, facile synthesis/preparation route, high ionic conductivity, and good performance, several issues are still present in each method. For example, the sol–gel and solvent-casting techniques have a lengthy drying step and the presence of residual solvent, both of which may be detrimental to battery performance and safety [7]. Furthermore, the solvent casting method can only be used to make thick electrodes, and the prolonged drying time may produce particle agglomeration or sedimentation, resulting in nonhomogeneous samples and reducing the electrolyte or electrode’s performance [13,14]. The evaporation of the solvent also creates gaps in the solid electrolytes and electrodes, which are unnecessary in all-solid-state batteries [15]. The solvent-free production of the polymerization-induced formation of IL-based gel shows drawbacks related to poor mechanical properties and the inability to form thin films.

## 3. Applications of IL-Based Gels

IL-based gel electrolytes are increasingly used in numerous applications such as lithium ion batteries, sodium batteries, fuel cells, solar cells, supercapacitors, microcapacitors, ionic actuators, electrochromic devices, and electrochemical sensors devices [15,24,73]. With advances in the technology of flexible electronic devices, such as smart electronics, stretchable devices, human body sensors, and internet of things, etc., IL-based gels have become a thriving research area. IL-based gel is a new kind of soft material with unique features such as high ionic conductivity, good electrode/electrolyte contact, and solid electrolytes ensuring safety, a large electrochemical window, and mechanical and thermal stability. These have gained much interest in a wide range of electronic applications, including next generation sustainable energy storage and conversion devices, microdevices, and flexible/stretchable devices. Recently, IL-based gels have been considered as a suitable replacement for traditional liquid and volatile organic electrolyte in electronic devices. The following section is dedicated to a description of some of the recent works dealing with the application of IL-based gels in the field of energy storage/conversion systems and electrochemical sensor and actuator devices.

### 3.1. Battery

The application of IL-based gel as a nonaqueous electrolyte and/or separator in batteries has been widely reported, especially in 2020; for example, in aluminum (Al) batteries, sodium (Na) batteries, calcium (Ca) batteries, organic batteries and, most of all, lithium (Li) batteries. Benefits such as a wide electrochemical window and relatively high ionic conductivity are the basic reasons for ionic liquid gel (ILG) composite modifications widely used as electrolytes in batteries [25]. Alongside liquid electrolytes, the prospect of IL-based gel applications in solid electrolytes has also been characterized and explored by Ashby et al. [27] who analyzed IL-based gel electrode interfaces in Li battery. The stretching properties that benefit electronic devices have also been the focus of research [74]. The general concept for using IL-based gels is as a cathode–anode interaction link.

The role of IL-based gels as electrolytes in batteries can be categorized into gel polymer electrolyte (GPE) or organic ionic gel (OIG), the latter being a new demonstration in 2020 by M. Bielejewski based on their previous report [75,76,77] in which he tested Li batteries and studied ion interactions and aggregation based on the effect of ionic conductivity on the liquid–solid interface of IL-based gels by optical spectroscopy and fast-field cycle diffusometry (FFC), nuclear magnetic resonance (NMR), and gradient pulsed (PFG) NMR methods. The difference between GPE and OIG is presented in Table 1. Further, M. Bielejewski introduced a glucopyranodise-derived monosaccharide that can bind a large amount of ionic liquid (IL) as a low molecular weight gelator (LMWG) in a basic IL-based gel system that is then dissolved in a mixed ionic solution, which may contain various types of ions or other charge carriers [77]. This dissolution requires the help of high temperatures to ensure everything is mixed successfully, but when all the components come together, it is enough to cool this mixture and turn it into a gel.

### 3.2. Li-Ion Batteries

Li batteries are still the most widely developed and commercialized. The nano approach involving cathode and anode materials, e.g., CNT anodes, silicon anode, and cathode nanoparticles, was revealed by Mekonnen et al. as the most promising for the development of Li-ion batteries [78]. The development of IL-based gel in this type of battery also varies according to the trend of the Li battery itself [24,79]. For example, IL-based gels, in order with most inventions, are found in Li-ion batteries, Li-metal batteries, Li–oxygen batteries, and Li polymer batteries.

**Figure 4:**
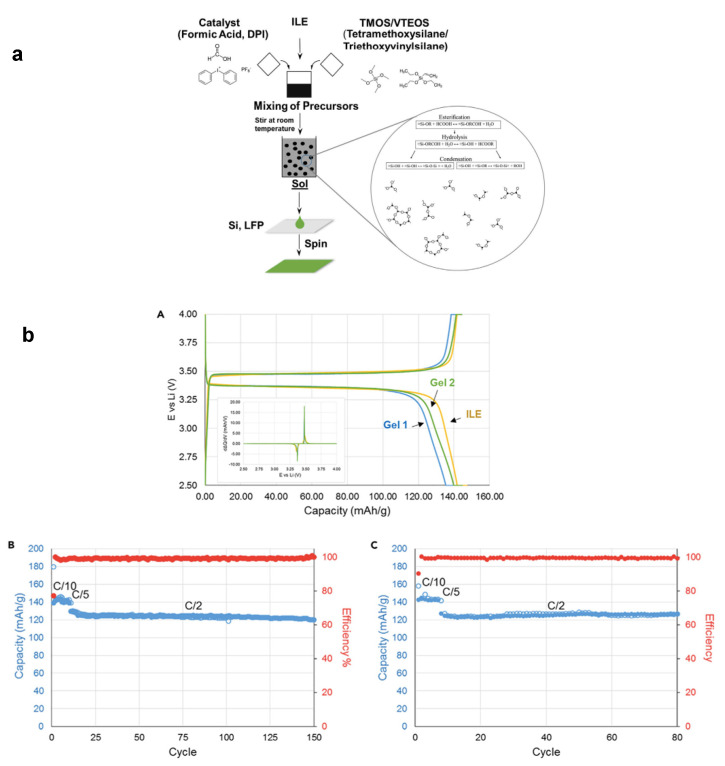
Schematic illustration of (**a**) spin-coating of TMOS/VTEOS IL-based gel electrolyte. (**b**) GV measurements of different electrolytes on LFP at C/10. Results are shown for gel 1 and gel 2, with the ionic liquid electrolyte (ILE) serving as a comparison (A). The inset shows the dQ/dV versus V curves extrapolated from the GV measurements. (**b**) Capacity variation with cycling for gel 1. Majority of the 150 cycles were at C/2 (B). Capacity variation with cycling for gel 2. Majority of the 80 cycles were at C/2 (C). Reprinted with permission from ref. [20].

In 2017, Ashby et al. [20] reported the development of two IL-based gel capabilities in two different cases. In the first case, the electrolyte was prepared as thin film, gel 1, by mixing and stirring TMOS/VTEOS and formic acid catalyst in IL, as shown in Figure 4a. The authors then made a Li iron phosphate (LiFePO_4_)/IL-based gel film/Li electrochemical device that operated at a capacity of 145 mAh g^−1^ at C/10 rate to ensure the ability of the sol to penetrate the LiFePO_4_ electrode; therefore, the resulting power density (203.15 W kg^−1^) and energy density (406.3 Wh kg^−1^) were higher compared to previously reported solid-state Li batteries [80]. Moreover, at increased C/2 rate, the device retains 96% of its initial capacity after 150 cycles (Figure 4b). In the second case, they examined the versatility of the IL-based gel for a new fabrication utilizing UV cross-linking synthesis by mixing TMOS, methanol, and BMIMTFSI IL containing 0.5 M LiTFSI (gel 2). The Li battery with LiFePO_4_ cathode, Li anode, and gel 2 electrolyte delivered a capacity of 124 mAh g^−1^ with an energy density of 403 Wh kg^−1^ and a power density of 201.5 W kg^−1^ at C/2 rate and impressive reversibility (80 cycles) with no virtual loss of capacity (Figure 4b). Ashby et al. again carried out an investigation in 2019 [27] and found the inhibitors of the reaction that they called ‘chemical attack’ and their solutions. In addition, this year, Guan et al. [81] also experimented with electron beam irradiation (EBI) to create stretchable cross-linked GPE (C-GPE). Poly(vinylidene fluoride-co-hexafluoropropylene) polymer film (P(VDF-HFP))/triallyl isocyanurate (TAIC) combined IL EMIMTFSI exhibited a tensile strength of 10.6 MPa, with almost full recovery after 100% stretching.

Contributing to the development of IL-based gels in Li-ion batteries, Ma et al. [82] revealed an increase in the ionic conductivity of a battery to 1.19 mS cm^−1^ that was assembled with a GPE membrane with 45.5% IL N-methyl-N-butyl-piperidine-bis(trifluoromethylsulfonyl) imide (NNBI). With cast, the membrane of GPE polyvinylidene fluoride (PVDF)/polyvinyl alcohol (PVA)/NNBI had a tensile strength of 24.11 MPa compared to PVDF/PVA with only 17.95 MPa. The capacities of 118 mAh g^−1^ and 136 mAh g^−1^ were achieved in the first and second discharge cycles.

A new, porous, cross-linked membrane GPE, used as a rechargeable Li-ion battery separator, was introduced in 2018 and contained ZnONP as pore-forming and filler materials [83]. The membrane is made from a mixture of P(VDF-HFP) and a cross-linked polymer of poly(ethylene glycol methyl ether methacrylate) (PEGMEMA), methyl methacrylate (MMA), and octavinyl-T8-silsesquioxane (OVPOSS), which were swelled by propylene carbonate (PC) and 18% 30nm ZnONP before being eluted by hydrochloric acid, resulting in micropores. The ionic conductivity of a Li-ion battery using this membrane is 1.4 mS cm^−1^ and the mechanical strength is 11.5 MPa.

In 2019, the GPE stretch film was again proposed by Yang et al. [49] for battery application with an elongation at break of 178%, a breaking strength of 6.65 MPa, and a high ionic conductivity of 0.80 mS cm^−1^. A used IL-based gel component is a mixture of P(VDF-HFP) poured on Pyr_13_TFSI and 0.6 g Li salt LiTFSI + 0.7 g IL Pyr_13_TFSI. In addition, the synthesis of a new family of chemically crossed IL-based gels was introduced by photopolymerization of branched aliphatic polyesters (HP-OH) with terminal acrylic groups [45]. Although quite complex, the results of this IL-based gel can achieve a mechanical strength up to 1.6 MPa, and are mechanically stable up to 200 °C, thermal up to 371.3 °C, and have an electrochemical window up to >4.3 V.

Additionally in 2019, Cheng et al. [30] paid attention to the flammable Li-ion battery electrolyte and then made a solution in the form of two types of quasi-solid-state IL-based gel electrolytes (IE-T and IE-B) using the fast sol–gel method and the structure of porous silica. A diamagnetic stirrer of 50 °C ethanol (EtOH) with Li trifluoromethanesulfonate (LiOTf) salt was used to prepare IE-T or LiTFSI salt to prepare IT-B with tetraethylorthosilicate (TEOS), then this was added to IL BMIMBF_4_. After that, it was printed and dried in a vacuum oven. IE-T has richer pores and a stable discharge capacity of 141 mAh g^−1^ at 0.1C for more than 100 cycles, whereas IE-B has low coulomb efficiency, poor reversibility, and high impedance. Based on that, IE-T is more secure.

In 2020, three Li-ion battery developments were reported by Chen et al., Yang et al., and Hu et al. [32,49,84]. Hu et al. [84] focused on improving the quality of refractory GPE with a simple and scalable phase inversion method. Moreover, poly(1,2-diethoxyethylimidazolium TFSI) (PDEIm)/P(VDF-HFP), which they tested directly on wax, also had rollable and scrunchable characters. LiFePO_4_/Li batteries using PDEim/P(VDF-HFP) show superior performance than those using a commercial Celgard 2325 separator saturated with a liquid electrolyte. Meanwhile, Chen et al. [32] made a new breakthrough 40 µm film mesoporous silica monolith matrix composite (nano-SCE) filled with BMPTFSI nonvolatile IL filler as a solid electrolyte option with Li-TFSI/TEOS salt. Furthermore, Yang et al. [49] paid attention to the thermal stability of Li-ion batteries by separating mechanical strength and ionic conductivity through the non-solvent induced phase separation (NIPS) method to produce an IL-based GPE gel P(VDF-HFP)-N-Methyl-2-Pyrrolidone (NMP)/IL Pyr_13_TFSI/LiTFSI with elongation at break of 178% and high thermal stability up to 220 °C. In the same year, Singh et al. [53] tested IL-GPE P(VDF-HFP)/PYR_13_FSI/LiTFSI on a LiNi_0.33_Mn_0.33_Co_0.33_O_2_ (LNMC) coated Li_2_CuO_2_ cathode to study the structural and cyclical stability of the cathode and found a twofold increase in its ionic conductivity compared to Yang et al. [49]. Another report submitted in 2020 by Matsuura et al. [85] shows the synthesis of tetra-arm poly(ethylene glycol) (TetraPEG) in triethylpentylphosphonium (P_225_) with Li-TFSI salt solution with an increased electrochemical window up to 4.2 V with 95% efficiency.

The following year, Wang et al. [37] introduced trimethylolpropane trimethylacrylate (TMPTMA)-based GPE polymerized with LiTFSI, dimethylcarbonate (DMC) and IL tributylmethylammonium bis(trifluoromethanesulfonyl) imide (TBMA-TFSI), which turned out to have the highest ionic conductivity compared to other LiTFSI-polymerized GPEs. Playing with widely developed materials, Ravi et al. [50,51,52] studied the composition dependency of LiTFSI and P(VDF-HFP) and Pyr_13_TFSI and found the maximal ionic conductivity of these electrolytes. A new introduction was also made this year by Hyun et al. to replace IL-based gel electrolytes using IL mixtures in the form of layered heterostructures because they can extend the window while maintaining high ionic conductivity [44]. This is a potential new development for further research.

Li–oxygen batteries are batteries that use the oxidation of Li at the anode and the reduction of oxygen at the cathode to induce an electric current. The development of the Li–oxygen GPE battery was initiated by Lind et al. [86] who incorporated the zwitterionic (ZI) sulfobetaine functional group into the IL EMIMTFSI supported by a polymer scaffold. ZI forms dipole–dipole interactions and ion–dipole interactions between them and the ILG, which promote the formation of physical polymer cross-links and the dissociation of IL cation/anion pairs, thereby increasing the ionic conductivity but still being able to maintain the thermal stability of the liquid. Then, in 2020, Woo et al. [87] explored ZI (N-methyl-N-(propane sulfonate) pyrrolidinium (MPSP)) in Li–Oxygen GPE batteries. They used a poly(methyl methacrylate) (PMMA) and the same IL containing ZI as previously reported.

In Li-metal batteries, only a few studies have been reported in the last 6 years: by Guo et al. in 2016 and Chen et al. in 2018 focusing on GPE; by Su et al. in 2019 with their improved organic–inorganic IL-based gel; and by Yang et al. in 2020 who summarized the IL-based gel electrolyte-based hybrid of polymer/inorganic [34]. The GPE made by Guo et al. [88] was carried out in two steps: membrane synthesis P(VDF-HFP)(SiO_2_-like silica airgel)-(butanone)(NMP) and membrane activation by immersion in diethyl ether for 24 h in 1 M LiTFSI solution in 1-ethyl- 3-methylimidazolium trifluoromethanesufonate/ethylene carbonate/propylene carbonate (EMITFSI/EC/PC), which produced a nonflammable GPE IL-based gel with lower strength than that proposed by Chen et al. [48]. They proposed a solution for the growth of Li dendrites by exploiting the strength of the ion–dipole interaction between the immobilized IL imidazolium (EMIMTFSI and GPE polysulfides (P(VDF-HFP)). This IL-GPE battery is auto rechargeable. The organic–inorganic electrolyte IL-based gel electrodes with semi-interpenetrated networks are designed for flame retardance, non-volatility, 83% capacity retention after 1000 cycles, and various other safety improvements because Su et al. considered traditional organic liquid electrolytes impractical and with low physicochemical properties. They combined Poly(IL), LiTFSI, GPOSS (cross-linker), and [EtO(CH_2_)_2_MMI]TFSI dissolved in acetone onto glass fiber (GF) [89]. Practicality is also the reason why, in the following year, Yang et al. [34] made a summary on a polymer/inorganic hybrid electrode (HE).

### 3.3. Na-Ion Batteries

**Figure 5:**
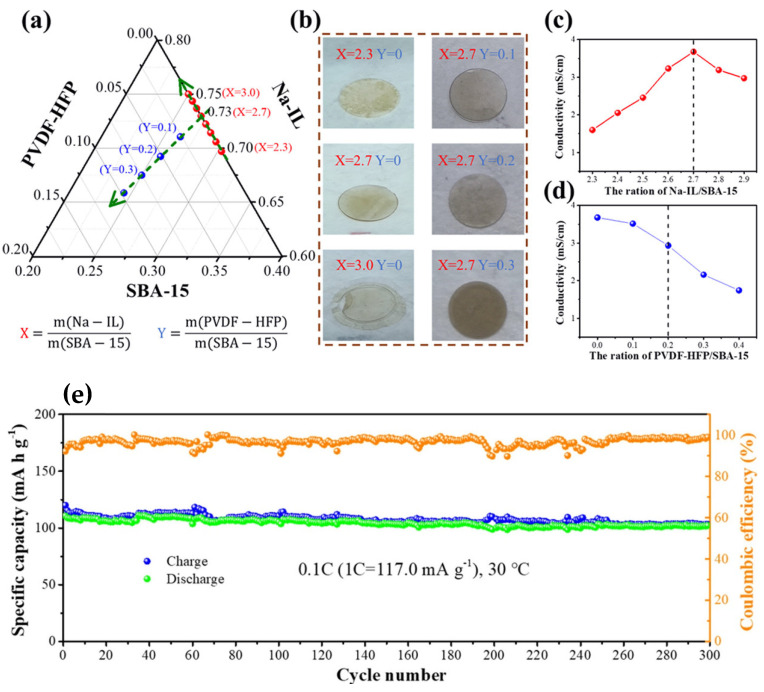
Proportion regulation and optimization of components in Na-IL/P(VDF–HFP)@SBA-15: (**a**) ternary phase diagram of Na-IL/PVDF–HFP@SBA-15 with different X and Y values; X, mass ratios of Na-IL/SBA-15; Y, mass ratios of PVDF–HFP/SBA-15; (**b**) digital photographs of Na-IL/PVDF–HFP@SBA-15 with X = 2.3, 2.7, 3.0 (fixed Y = 0) and Y = 0.1, 0.2, 0.3 (fixed X = 2.7); (**c**,**d**) ionic conductivity of Na-IL/P(VDF–HFP)@SBA-15 corresponding to different ratios of Na-IL/SBA-15 and different ratios of PVDF–HFP/SBA-15 at room temperature, with fixed Y = 0 and X = 2.7, respectively; (**e**) cycle performance of Na| Na-IL/PVDF–HFP@SBA-15|NVP at 0.1 C (30 °C). Reprinted with permission from ref. [69].

Following the good fortune of Li batteries, a new breakthrough, the rechargeable Na-ion battery, promises a wider commercialization as according to the research community and industrial entities such as Electrochemical Energy Storage Systems (EESS) [90,91]. Future perspectives [92] and suitable materials [90] for Na batteries have also been explained previously. Starting in 2020, several IL-based gel breakthroughs in Na-ion batteries were also developed, one of which was by Gao et al. using polymer-based IL-based gel [69] and Zheng et al. using inorganic-based IL-based gel [93]. Gao et al. reported an electrochemical enhancement of high safety IL-based gels using Pyr_13_FSI comprising SiO_2_-based mesoporous sieve (SBA-15) host as an ionic conductor due to its high conductivity, Na bis(trifluoromethane)-salt sodium sulfonymide (NaTFSI) because of its larger anionic radius than other Na salts, and 12 P(VDF-HFP) as a binder due to its high dielectric constant. P(VDF-HFP) increases the mechanical strength of Pyr_13_FSI containing NaTFSI when ground into nanoparticles with short-range ordering mesoporous structures, thereby increasing the storage space in the battery. Figure 5a,b shows the proportion regulation and optimization of components in Na-IL/PVDF–HFP@SBA-15. The ionic conductivities of the IL-based gel electrolytes are displayed in Figure 5c,d. As presented in Figure 4e, with the Na ion battery consisting of Na_3_V_2_(PO_4_)_3_ (NVP) cathode and a metallic Na anode with Na-IL/P(VDF-HFP)@SBA-15 IL-based gel electrolyte, an initial discharge specific capacity of 110.7 mA h g^−1^ was obtained, and the cell retained 92% of its initial capacity after 300 operational cycles at 30ºC. In contrast to solid-state Na ion batteries, Zheng et al. demonstrated a planar Na-ion micro-battery using a NaBF_4_-based IL-based gel electrolyte, showing a high ionic conductivity of 8.1 mS cm^−1^, with a Na titanate (NTO) anode and a Na vanadate phosphate (NVP) cathode. The cycles of these Na micro-batteries last longer than those reported by Gao et al. and can be discharged and charged to levels of 100 C in just 15–20 s.

### 3.4. Al-Ion Batteries

Al-ion batteries are a low-cost and rechargeable high-energy density battery option considering their abundance, high resistance to oxygen and moisture, and the four times higher volumetric capacity of Al compared to Li [94]. Das and Lahan [95] have summarized a number of proposed electrochemical reactions for various Al-ion cells, while Elia et al. [96] reported their prospective related views on Al-battery technology. The disadvantages of Al-ion batteries, such as relatively short shelf life [97], are overcome by electrodeposition of Al in the form of a polymer-based IL-based gel of 35 mol-% 1-ethyl-3-methylimidazolium chloride 65 mol-% Al chloride (EMIMCl-AlCl_3_) with polymer gelification of polyethylene oxide (PEO), which was reported by Schoetz et al. [98] who focused on Al-conductive polymer battery (ACP). Theoretically, they used a Lewis acid (at the anode) and a neutral IL electrolyte (at the cathode) with a Lewis neutral IL-based gel at both electrodes. For the first time, the deposition of Al reached a potential stability window of 5 V without any dendritic growth, with 60% coulombic efficiency potentially able to be further increased.

### 3.5. Ca-Ion Batteries

Ca-ion batteries are a prospect for a broad field of research, and there is much scope for the development of electrodes and electrolytes that enable stable long-term battery operation [99]. Due to this promising alternative to post-Li batteries that are more cost-effective and can be applied on a large scale, various designs of Ca batteries have been devised to facilitate research [100,101]. For the first time, liquid GPEs were used as electrolytes and separators in Ca-ion batteries at room temperature. Biria et al. [40] prepared an electrolyte solution of Ca perchlorate salt (Ca(ClO_4_)_2_), Ca-tetrafluoroborate (Ca(BF_4_)_2_), and Ca bis-(trifluoromethylsulfonyl)imide (Ca(TFSI)_2_) dissolved in 1-ethyl-3-methylimidazolium trifluoromethanesulfonate (EMIMTFMS) to mix with 2.5% poly(ethylene glycol) diacrylate-photoinitiator camphorquinone (PEGDA-CQ) and 1.5% 4-octyloxyphenyl)phenyliodonium hexafluoroantimonate (4OPP-HFA) as a photoinitiator system, and then injected them into a Teflon ring cell and exposed them to a light emitting diode (LED) to form a gel. They reported ionic conductivity at room temperature between 10^−4^ and 10^−3^ S cm^−1^, stability of up to 4 V, a cationic transfer rate of 0.17, high thermal stability up to 300 °C, and an initial discharge capacity of 140 mAh g^−1^.

**Figure 6:**
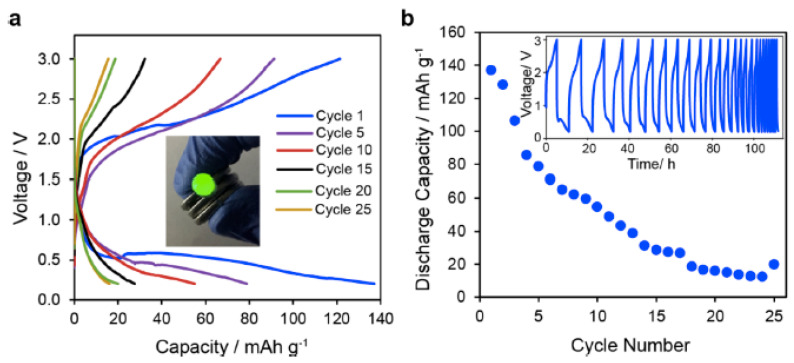
Preliminary battery operation using the IL–GPE in a Ca_3_Co_4_O_9_–V_2_O_5_ full cell. (**a**) Galvanostatic charge and discharge curves, attaining discharge capacities reaching 140 mAh/g in the first cycle. The inset shows a stack of three–coin cells powering a small green LED. (**b**) Plot of discharge capacity vs. cycle number. The inset shows charge–discharge voltage over 25 cycles. Reprinted with permission from [40].

They further constructed a full cell battery consisting of a vanadium oxide (V_2_O_5_) anode and a calcium cobalt oxide (Ca_3_Co_4_O_9_) cathode, particularly for the case of Ca(TFSI)_2_ salt. The battery offered first cycle charge and discharge capacities above 120 mAh/g and an open-circuit voltage of 1.2 V (Figure 6a). In addition, the battery was charged and discharged at operating cell voltages from 3 V (cutoff) to 5 V for 25 operational cycles (Figure 6b).

Organic batteries are proclaimed to be the lifestyle of the future because they are environmentally friendly, simple, inexpensive, and can be charged and emptied multiple times at high faradaic efficiencies with no signs of degradation [102]. The trends and developments in organic batteries have been summarized by Friebe et al. [103]. Roll-to-roll printable GPE was also developed for all-organic batteries. Muench et al. [46], placed IL-based gel in the form of 370 GPE microfilms of BMIMTFSI and a polymer matrix of poly(ethylene glycol) methylether methacrylate (mPEG-MA) directly onto a solid-state battery electrode, and then polymerized using UV-polymerization to create a stable film. The advantage of this process is that it does not require a solvent. They tested with a poly(2,2,6,6-tetramethyl-4-piperidinyl-N-oxyl methacrylate) (PTMA) cathode and a poly(2-vinyl-11,11,12,12-tetracyano-9,10-anthraquinondimethane) (poly(TCAQ)) anode and found positive reports when this IL-based gel was applied as an electrolyte and separator in organic solid-state batteries.

To make it easier to compare the applications of various IL-based gels on various batteries, the reports are arranged in Table 2. Formulating IL-based gel electrolytes with a large electrochemical window and ionic conductivity is an important research orientation for obtaining high-performance batteries. On the other hand, flammability, membrane solid IL-based gel, and porous coated electrodes are also important research orientations because the selection of IL and the structure of the electrodes used also significantly affect the electrochemical performance of the battery. From the table, it is known that the potential ionic conductivity of the Na micro-ion battery is far higher other batteries, so it is necessary to try other IL-based gels and compare them with this type of battery. Moreover, HE and OIG were only demoed in 2020 and are worth checking out as a GPE alternative given their simpler build. Therefore, the discovery of new immobilized matrices in IL-based gel composites and new electrode structures is still needed to produce better battery specifications.

### 3.6. Supercapacitors

Electrochemical capacitors, commonly referred to as supercapacitors (SCs), are considered as promising energy storage devices that store electrical energy at the interface between the electrode and electrolyte. Recently, SCs have attracted increasing attention because of their high power density (~10 kW kg^−1^), outstanding cyclic stability (~1,000,000 cycles), fast charge–discharge rate, and superior safety while keeping a reasonable energy density [104]. Micro-supercapacitors (MSCs) offering high-energy and power performance are known as a class of supercapacitors with a miniaturized configuration. SCs and MSCs are emerging as high-performance electrochemical energy storage and clean renewable energy generation devices that supply power for various electronic devices, including hybrid vehicles, portable electronics, military devices, space equipment, next-generation electric cars, microdevices, and internet of things. Based on the energy storage principle, SCs can be classified into two types, i.e., electric double layer capacitor (EDLC), where charge is stored through fast ion adsorption at the electrode/electrolyte interface, and pseudocapacitor, which stores electrical energy by reversible redox reactions at the surface of the electrodes. Traditionally, liquid electrolytes have been used in SCs, which is often associated with several drawbacks such as narrow potential window, limited cycle stability, and possibility of leakage. IL-based gel electrolytes exhibiting a wide potential window, less volatility, high conductivity, and good mechanical strength are considered an advantageous alternative to typical liquid electrolytes [105].

To date, efforts have been made towards the fabrication and design of highly efficient SCs and MSCs with IL-based gel electrolytes [106,107,108]. Among the various SCs, lithium ion capacitors (LICs) are reported to be a promising energy storage device that combines the high energy density of batteries and high power density of SCs [109]. Recently, carbon-based materials such as carbon black, activated carbon (AC) CNTs, graphene, and reduced graphene oxide (rGO) have also been used intensively as electrodes in SCs due to their high specific area and remarkable conductivity [110]. In 2015, Ujjain and coworkers fabricated an SC using modified rGO electrodes with IL-based gel electrolyte consisting of EMIMBF_4_ IL and P(VDF-HFP). The SCs showed a high specific capacitance (*C*sp) of 242 F g^−1^ at 5 mV s^−1^ and a charge/discharge stability up to 12,000 cycles [111]. By using a BMPTFSI/PMMA electrolyte and graphene electrode, Tamilarasan et al. assembled a high-performance SC possessing a specific capacitance, energy density, and power density of 83 F g^−1^, 25.7 Wh Kg^−1^, and 35.2 kW kg^−1^, respectively. [112]. Similarly, Lee and colleagues reported the construction of SCs using P(VDF-HFP)/EMIMTFSI electrolyte and polydimethylsiloxane/CNT electrodes, which exhibited a superior capacitance retention of 96.6% for up to 3000 cycles [113].

In 2018, Taghavikish et al. introduced a (1,4-di(vinylimidazolium)butane bisbromide) (DVIMBr) and 2-hydroxyethylmethacrylate (HEMA)-based IL-based gel electrolyte in 1-butyl-3 methylimidazolium hexafluorophosphate (BMIMPF_6_) IL solvent for EDLC application [105]. The supercapacitor with IL-based gel electrolytes showed promising performance with a specific capacitance of 1.5–3 mF cm^−2^, as revealed from electrochemical tests. In the same year, Liu et al. reported an aligned IL-based gel prepared by UV-copolymerization of N,N-dimethylacrylamide (DMAA) and BMIMBF_4_ IL for high-temperature SCs with a specific capacitance of 176 F g^−1^ at 25ºC using carbon nanocage electrode materials [114]. Meanwhile, Kim et al. polymerized poly(hydroxyethyl methacrylate-co-poly (ethylene glycol) dimethacrylate) copolymer (PHEMA-co-PEGDMA) in EMIMBF_4_ under UV irradiation to obtain an IL-based gel electrolyte for flexible SCs with commercially activated carbon (MSP-20) as electrode materials [115]. The synthesized IL-based gels exhibited an ionic conductivity of 12.27 mS cm^−1^, which is comparable to pristine ILs. The electrochemical test for SCs’ cells showed a good performance by possessing a specific capacitance of 163.17 F g^−1^ and retaining 59% of its initial capacitance. A study published in 2017 demonstrated the preparation of a hybrid IL-based gel electrolyte consisting of epoxy-functionalized POSS, amine-terminated polypropylene glycol, and 1 M LiTFSI in BMPTFSI IL for high-temperature LICs [109]. The authors further evaluated the cycling performance of the hybrid IL-based gel electrolyte using CR-2032 coin-type cells consisting of a separator, Li metal, and AC electrode at a constant current density of 0.2 C/0.5 C at 80 °C. The as-designed cell with IL-based gels exhibited excellent cycling behavior with >97% coulombic efficiency. In the following year, 2018, Jiao and colleagues developed quasi-solid-state Li-ion-type asymmetric supercapacitors (ASCs) based on *T*-Nb_2_O_5_/rGO nanohybrids for the anode, LiTFSI-EMIMBF_4_ mixture for the electrolyte, and a P(VDF-HFP)/EMIMBF_4_/LiTFSI IL-based gel separator. They suggested that the *T*-Nb_2_O_5_/rGO electrodes have great potential for Li^+^ storage [116]. Recently, in 2020, Yao et al. reported optimized solid lithium ion-type ASCs based on a TiNb_2_O_7_@ MoS_2_/C anode, AC cathode, and the IL-based gel electrolyte composed of EMIMTFSI IL, LiTFSI salt, P(VDF-HFP), and SiO_2_ [54]. From electrochemical performance, the maximum energy density and power density of lithium ion-type ASCs were estimated to be 147.2 Wh kg^−1^ and 2470.5 W kg^−1^, respectively, at 60 °C. Similarly, Li et al. developed TiNb_2_O_7_@carnon nanofiber(CNF)-based LICs exhibiting a superior energy density of 397.3 μWh cm^−2^ with an EMIMBF_4_/P(VDF-HFP)/LiTFSI IL-based gel as the separator [117]. Meanwhile, Pazhamalai and coworkers used a P(VDF-HFP)/tetraethylammonium tetrafluoroborate (TEABF_4_) ion gelled, electrospun PVDF/NaNbO_3_ nanofibrous mat separator and a 2D-MoSe_2_ nanosheets electrode for the construction of self-charging SCs [118]. On the other hand, Liu et al. constructed a new NiO/rGO//AC-based LIC using P(VDF-HFP)-EMIMBF_4_-LiTFSI IL-based gel electrolyte obtained via the solution casting method [104].

Besides LICs, in 2020, Xing et al. introduced solid-state sodium ion capacitors using a MoS_2_/CNT working electrode and sodium foil counter-electrode in P(VDF-HFP)/EMIMBF_4_/NaTFSI IL-based gel electrolyte [57,58]. The sodium ion capacitors showed profound performances with an outstanding cyclic stability of 81.2% after 8000 cycles at above 30 °C.

**Figure 7:**
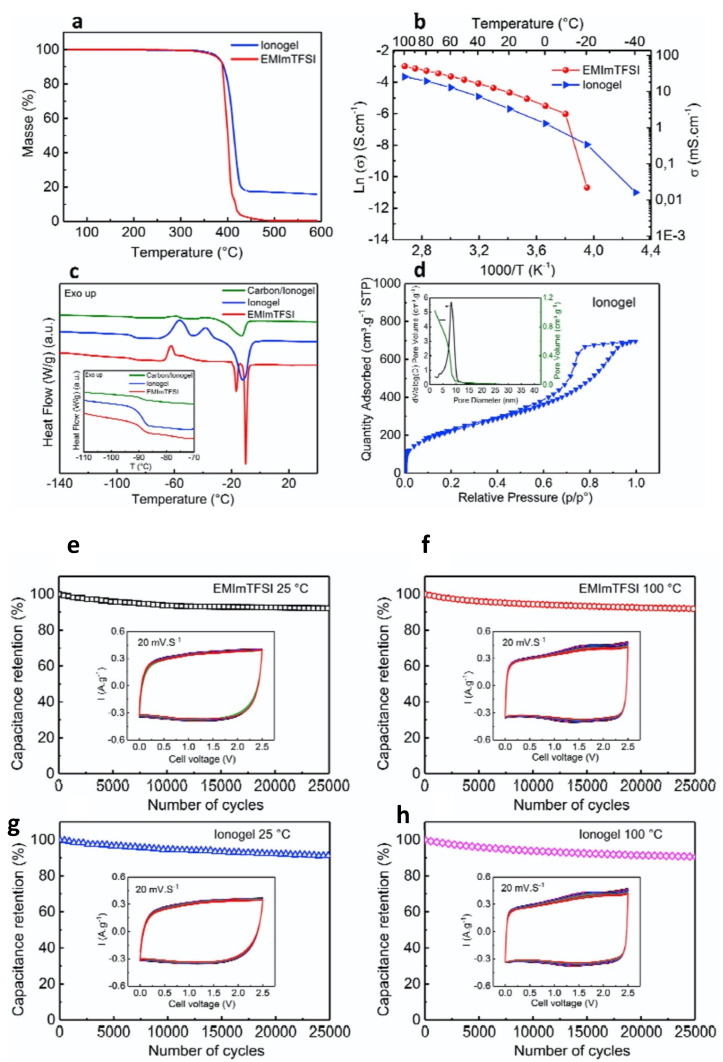
(**a**) TGA showing the thermal stability of the pristine IL and of the IL–based gel; (**b**) conductivity versus temperature; (**c**) DSC profiles of the IL and of the IL-based gel; (**d**) N_2_ adsorption/desorption isotherm; inset: BJH pore size distribution for the IL-based gel after IL extraction. Galvanostatic cycling with potential limitation (GCPL) data at room temperature (**e**,**g**) and 100 °C (**f**,**h**) for EDLCs using IL (**a**,**b**) and IL-based gel electrolytes (**c**,**d**); insets: CV at 20 mV s^−1^ recorded after every 1000 GCPL cycles; at the 25,000th cycle, the charge efficiency is 78, 85, 73, and 83% for e, f, g, and h, respectively. Reprinted with permission from ref. [29].

Meanwhile, Asbani et al. assembled high-temperature EDLCs using carbon electrodes and a sol-made IL-based gel composed of EMIMTFSI, TMOS, and DMDMS [29]. From Figure 7a, the TGA thermograms show that the mass of IL-based gel remains unchanged until 350 °C. A complete degradation observed at about 470 °C and 440 °C for EMIMTFSI and IL-based gel, respectively, indicated that IL-based gel exhibits favorable thermal stability. The IL-based gel displayed the ionic conductivity of 4 mS cm^−1^ at 20 °C, which was found to increase to up to 26 mS cm^−1^ at 100ºC (Figure 7b). The conductivity values of the sol–gel-derived IL-based gel were close to those of corresponding IL between 20 °C and 100 °C. The glass transition temperature (Tg) of EMIMTFSI was reported to be −70 °C, decreasing only slightly with silica content from −88 °C to −90 °C, as shown in the DSC profile (Figure 7c). From BET isotherm (Figure 7d), the surface area of the IL-based gel was calculated to be 800 ± 10 m^2^ g^−1^. The pore size distribution was about 8 nm, as obtained by the Barett–Joyner–Halenda (BJH) method. Figure 7e,f shows the cyclic voltammograms recorded for the EDLCs at 20 mV s^−1^ after every 1000 cycles for up to 25,000 cycles at room temperature. The solid-state EDLCs displayed a super high cyclic stability, with about 92% capacity retention after 25,000 cycles. In a previous report, Ortega’s group showed the electrochemical performance of IL-based gel-based EDLCs with a capacitance retention of 88% after 2000 cycles, which was lower than the EDLCs reported by Asbani et al. [29,119].

In 2017, Lé et al. utilized an IL-based gel composed of propylene carbonate (PC) and butyltrimethylammonium bis(trifluoromethylsulfonyl)imide (N_1114_ TFSI) IL (50:50%wt) as an optimal electrolyte for symmetric MSCs based on silicon nanowire (SiNW) using a large and stable cell voltage of 3.5 V. After 3 × 10^6^ galvanostatic charge–discharge cycles at room temperature, the capacitance of the MSCs was reported to be 150 mF cm^−2^ [120].

Meanwhile, in 2020, Asbani et al. developed and tested 3D MSCs using MnO_2_ electrodes and a sol–gel derived IL-based gel containing PVDF in DMF and EMIMTFSI [121]. The IL-based gel-based MnO_2_ MSC delivered excellent cycle life over 30,000 cycles. In a previous report, Zhou’s group described the fabrication of fluorine-modified graphene (FG)-MSCs using EMIMBF_4_/P(VDF-HFP) IL-based gel electrolyte, which offered a high energy density of 59 mWh cm^−3^ [122]. Table 3 summarizes some recent developments in IL-based gel-based SCs for next-generation energy storage devices.

### 3.7. Fuel Cell

For decades, enormous efforts have been made in the development of a high-performance fuel cell (FC) for new clean energy generation and storage devices. Fuel cells directly generate electricity by the electrochemical oxidation of H_2_ at the anode and the reduction of O_2_ into water at the cathode. Although the application of IL-based gel electrolytes in FC has been limited until now, several research groups attempted to design stable FC with ionic liquid-based electrolytes [106]. In 2018, by using the sol–gel approach, Chang et al. developed a TEOS/trimethylammonium methanesulfonate (ES) IL-based gel electrolyte for proton exchange membrane fuel cell (PEMFC) application [132]. The as-prepared IL-based gel showed a superior ionic conductivity of 38.5 mS cm^−1^ at 120ºC under a nonhumidified condition. The electrochemical results showed that the PEMFC constructed by sandwiching TEOS/ES IL-based gel electrolyte between two gas diffusion layer (GDL) electrodes possessed a maximum power density of 43.3 mW cm^−2^ at a current density of 92 mA cm^−2^ at 30ºC under a nonhumidified condition. Later, by using the solution casting method, Rao and his team prepared a novel proton conducting IL-based gel consisting of N,N-diethylmethylammonium triflate (DEMATf) and poly(diallyldimethylammonium) bis(trifluoromethanesulfonyl) imide (PDADMATFSI) for FC applications [133]. The synthesized DEMATf/PDADMATFSI IL-based gel with 60% DEMATf exhibited an enhanced ionic conductivity of 5.8 mS cm^−1^ at 100ºC, which was higher than that of commercial Nafion (0.2 mS cm^−1^) at 120 °C.

**Figure 8:**
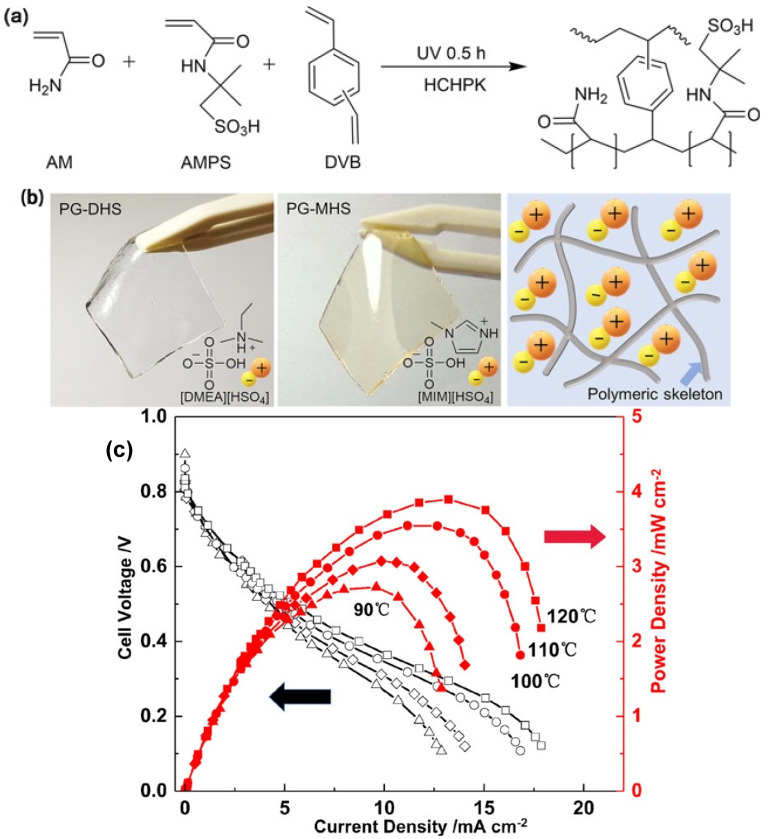
(**a**) Synthetic route of the polymeric skeleton, (**b**) the illustration of PG-DHS and PG-MHS IL-based gels, and (**c**) polarization curves of the H_2_/O_2_ fuel cell prepared from PG-MHS at temperatures from 90 to 120 °C. Reprinted with permission from ref. [134].

Recently, in 2021, Zou’s research group used the UV-polymerization technique to fabricate a protic IL-based gel from acrylamide (AM), 2-acrylamide-2- methyl-propanesulfonic acid (AMPS) monomers with a divinylbenzene (DVB) cross-linker and 1-hydroxycyclohexylphenylketone (HCHPK) initiator in dimethylethylammonium hydrogen sulfate (DMEAHSO_4_) or 1-methylimidazoilum hydrogen sulfate (MIMHSO_4_) IL (Figure 8a,b) [134]. The obtained IL-based gels in DMEAHSO_4_ and MIMHSO_4_ were denoted as PG-DHS and PG-MHS, respectively. The PG-DHS and PG-MHS possessed a high ionic conductivity of 17.1 and 10.8 mS cm^−1^, respectively, at 120 °C. To construct FC electrodes, a suspension of Pt/C particles and Nafion in isopropanol and water was sprayed onto the GDL electrode. Finally, the H_2_/O_2_ FC was assembled at 120 °C by sandwiching the protic PG-MHS IL-based gel sheet accommodated in a silicone rubber gasket between the GDL electrodes. From the polarization curve, as shown in Figure 8c, a peak power density of 3.9 mW cm^−2^ can be achieved in PG-MHS FC at 120 °C.

### 3.8. Electrochromic Device

Electrochromic devices (ECDs) are finding widespread architectural applications in energy-saving smart windows, automotive mirrors, privacy glass, displays, electronic paper, military camouflage and so on [135,136,137]. ECDs undergo reversible color change due to electrochemical oxidation reduction in response to electric field. Typically, an ECD consists of a soft substrate, conductive electrode, electrochromic material layer, ion storage layer, and electrolyte. The electrolyte plays a crucial role in the coloration/bleaching efficiency of the ECDs. In modern ECDs applications, compared to liquid electrolytes, ionic liquid based gel electrolytes or ionogels have attracted immense attention owing to their outstanding ionic conductivity and improved mechanical and electrochemical stability [135]. In this section we introduce some of the latest developments in ionic liquid based gel electrolytes for ECDs application. 

In 2021, Marija et al. used a sol-gel derived ionogel comprised of trialkoxysilyl-functionalized 1,14-bis(3-(3-(3-methoxysilyl)propyl)imidazolium 1-il)-3,6,9 trioxa undecan diiodide ((EO)_3_[TMSPIm^+^I^−^]_2_) and 1-methyl-3-propyl imidazolium iodide (MPIm^+^I^−^) ionic liquids in acetic acid [138]. These redox electrolytes having various iodine contents with ionic conductivity in the range of about 1 mS cm^−1^ were tested in hybrid ECD for 11,000 operational cycles. The ECDs exhibited similar optical modulation and was approximately 30–35% at 634 nm. Previously in 2017, Tang and coworkers prepared a gel electrolyte by dissolving PMMA, 1 M LiClO_4_ in propylene carbonate and EMIMBF_4_ [139]. The ECD assembled as glass/fluorine doped tin oxide (FTO)/WO_3_/PMMA–EMIMBF_4_ composite electrolyte/FTO/glass showed an enhanced electrochromic performance with a reduced anodic reaction potential. The as designed ECD was therefore found to be bleached easily at a smaller external voltage. Meanwhile, Santiago et al. designed flexible ECD displays using P(VDF-HFP) with butyl-methylammonium bis(trifluoromethanesulfonyl)imide (N_1114_TFSI) (79% wt.) which showed high transparency with 83% transmittance and ionic conductivity of 1.06 mS cm^−1^ [140]. In 2018, Yun et al. fabricated an ECD by sandwiching a ionic gel containing P(VDF-HFP) and BMPTFSI or BMIMBF_4_ between ITO-coated glasses and the devices were tested with two electrochromic materials, diheptyl viologen bis(hexafluorophosphate) [DHV(PF_6_)_2_] and monoheptyl viologen hexafluorophosphate [MHV(PF_6_)] [141]. The MHV^+^-based devices exhibited high color purity, large transmittance, and good coloration efficiency compared to that of DHV^2+^ -based ECDs. It was expected that MHV+ can act as a solid state ECD electrolyte while providing magenta (reddish) pixels for display applications. Recently in 2021, More et al. reported construction of an ECD utilizing a metal-organic frameworks (MOFs) and 1-hexyl-[4,4′ -bipyridin]-1-ium bis(trifluoromethane sulfonyl) imide [MHV][TFSI]-based hybrid ionogel electrolyte [142]. The prepared ECD showed a remarkable stability upto 2000 cycles with high coloration efficiency of 99.14 cm^2^/C and fast coloration time of 4.35 s and bleaching time of 7.72 s.

## 4. Challenges and Future Opportunities of IL-Based Gel

**Figure 9:**
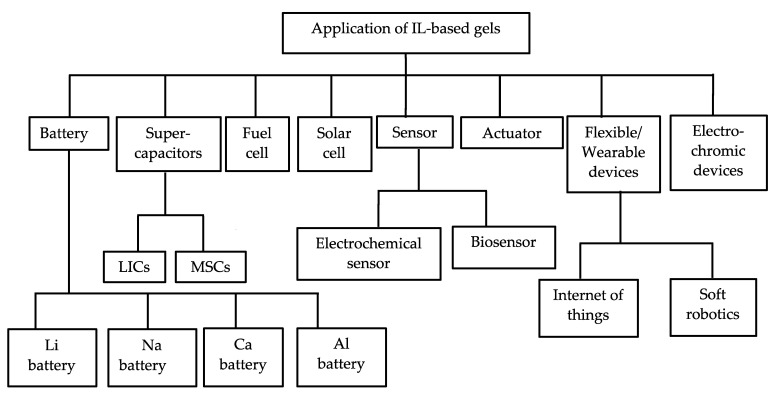
Potential application of IL-based gels in electrochemical devices.

IL-based gels offer a combination of simple preparation, a wide electrochemical potential window, high ionic conductivity, and enhanced stretchability. However, IL-based gels often suffer from poor mechanical strength such as stiffness, pinholes, and low toughness. In addition, some IL-based gels prepared from hydrophilic ILs or polymers are unstable in a humid environment. By contrast, IL-based gels composed of hydrophobic ILs possess excellent voltage stability and air and water resistance and are noncorrosive to metal electrodes. Although the conductivity of IL-based gels is lower than that of pristine ILs, researchers are exploring IL-based gel electrolytes as stable alternatives for Li batteries, SCs, actuators, sensors, wearable electronics, and internet of things. Meanwhile, MSCs that can be directly placed on chips are widely used as a power source for micro-/nano-electronic devices by instantly providing effective peak power. The urgent need for trendy, portable, smart, wearable devices and real-time communication devices has led scientists to design and commercialize IL-based microelectronics in recent years. Thus, the future possibilities offered by IL-based MSCs are worth exploring. The potential and future opportunities of IL-based gel application in electrochemical devices are shown in Figure 9.

To date, Li batteries with IL-based gel are the most reported. This opens up great opportunities for IL-based gel modifications to other types of batteries. For SCs, power density and energy density are the critical parameters. Most of the existing SCs suffer from lower energy density compared to the Li battery. However, Li batteries often encounter a drawback of low power density, short lifecycles, and low safety. Advanced hybrid capacitors (HCs) are a new class of energy storage system consisting of a battery-type anode and a capacitive cathode that have attracted great attention by researchers and have begun to develop rapidly. In HCs, the anode undergoes redox reactions or reversible lithiation/delithiation, whereas the cathode stores energy through the electric double-layer capacitance. HCs can offer the long lifespan of SCs, the high energy density of batteries, and the longer cycling life of electronic devices [143]. The ever-increasing demand for power density and energy density systems in hybrid vehicles has necessitated the development of advanced HCs in recent years. Hence, the environmentally friendly IL-based advanced hybrid supercapacitors have great possibilities for next-generation energy storage devices [143,144]. Currently, IL-based gel electrolytes are also being used in solar cell applications [145,146]. Usually, IL-based gels are applied as a solid-state electrolyte or solid polymer electrolytes for energy storage device applications. The appropriate choice of electrode materials, IL, and the matrix of the IL-based gel can shed light on developing and exploiting novel smart and sustainable energy storage devices and more geometrically complex electronics. Besides electrochemical device application, another great potential of IL-based gels is to be used in electrochromic devices and as a heterogenous catalyst and biocatalyst [16,147,148]. IL-based gels as biocompatible materials were found to be effective in minimizing drug degradation and loss in drug delivery systems [16].

Intelligent structural design and optimization-based performance are required, for which it is important to carry out extensive and thorough experimentation. Furthermore, complex synthesis routes and device preparation processes need to be avoided. Digitalization of synthetic routes is required to create touchless automatic fabrication, such as via 3D printing or additive manufacturing. Some works on 3D printable IL-based gels and composites have been reported [149,150,151,152], however, progress is very limited, mainly due to the limited compatibility of IL and monomeric systems and suitable polymerization processes for 3D printing. The difficulties in this area can be overcome via extensive research and multidisciplinary collaboration. Simultaneously, to ensure a world with a better future, it is high time to create IL-based gels that are both biologically safe and easily degradable.

Additionally, to significantly improve the performance of IL-based gels, a gel system with a high dissociation constant at low temperature is required to be developed. Some possible approaches can be taken, such as developing novel ILs and IL-based monomers, mixed ILs, and eutectic salts containing two or more types of ILs and utilizing these in gel systems. We hope that this perspective will lead to a better understanding of ion gels and their applications and close the gap between the related multidisciplinary fields. In addition to the LIBs and SCs addressed in this study, it is envisaged that as IL research advances, they will be utilized for future energy storage and conversion devices, such as multivalent ion batteries, metal air batteries, and high-efficient fuel cells.

## 5. Conclusions

The synthesis approaches of IL-based gels using sol–gel and polymerization processes are covered in the first section of this review paper. The later part of the paper focused on the applications of IL-based gels, such as their use in batteries, fuel cells, and supercapacitors. The use of ILs and IL-based gels and polymers in energy storage and conversion devices is continuing and must continue in the future to improve the electrochemical performance and stability of energy storage devices. In the final section, we explored the obstacles and the potentials that we believe can act as a catalyst for researchers to pursue further advanced practice of IL-based materials in energy sectors.

## Figures and Tables

**Table 1:** The difference between GPE and OIG.

GPE	OIG
+ solid or semisolid phase + high mechanical strength + wide temperature range - complicated manufacturing process - non-recyclable	+ solid-like phase + higher conductivity - medium temperature range + easy manufacturing process + recyclable + eco-friendly

**Table 2:** Electrochemical performances of IL-based gel electrolytes for applications in batteries.

Battery	Cathode	IL-Based Gel	Anode	Preparation Process	Lifecycles	Stability Window	Power and Energy Density	Ionic Conductivity	Refs.
ACP battery	EMIMCl-AlCl_3_ (neutral)	EMIMCl-AlCl_3_-PEO	Lewis acid	Magnetic stirrer	260 mAh g^−1^, reaches its maximum at 50 cycles	≤4-≤5 volt	598 Wh/kg and 374 W/kg	-	[98]
Na-ion battery	Na_3_V_2_(PO_4_)_3_	P(VDF-HFP)/Pyr_13_FSI-NaTFSI	Metallic Na	Mechanical ball milling, roller pressing	101.8 mAh g^−1^, 300 cycles	Up to 4.8 volt	-	2.48 mS cm^−1^ at 30 °C	[81]
Na-ion micro-battery	Na_3_V_2_(PO_4_)_3_	NTO/NaBF_4_-IE/NVP	NTO	Electrochemical deposition	30.7 mAh cm^−3^, 3000 cycles	1–5 volt	4 W/cm^3^ and 55.6 mWh/cm^3^	0.18 mS cm^−1^ at 30 °C	[93]
Ca-ion battery	Ca_3_Co_4_O9/carbon/PVDF binder in NMP solvent	EMITFMS/PEGDA-CQ/4OPP-HFA	Vanadium oxide/carbon/PVDF binder in pure copper foil (cleaned with NMP)	Light emitting diode	140 mAh g^−1^, 25 cycles	≤3 volt	-	Between 1 and 0.1 mS cm^−1^ at up to 300 °C	[40]
Solid-state all-organic battery	PTMA	BMIMTFSI/mPEG-MA	Poly(TCAQ)	UV-initiated polymerization	17 mAh g^−1^, 1000 cycles	1 V	-	Up to 0.1 mS cm^−1^ at room temperature	[46]
Li-ion battery	LiFePO_4_	TMOS/VTEOS	Li	Spin-coating	125 mAh g^−1^, 150 cycles	4 V	203.15 W/kg and 406.3 Wh/kg	-	[20]
	LiFeO_4_	PVDF/PVA/NNBI	Li	Casting method	104.6 mAh g^−1^, more than 95% efficiency after 99 cycles	4.2–2.5 V	-	1.19 mS cm^−1^	[82]
	LiFePO_4_	P(VDF-HFP)/TAIC/EMIMTFSI	Li	EBI	Max 64.1 mAh g^−1^, still 30.6 mAh g^−1^ after 30 cycles	2–4.5 V	-	-	[81]
	LiFePO_4_	P(VDF-HFP)(Pyr_13_TFSI)/(LiTFSI)(Pyr_13_TFSI)	Li	Nonsolvent induced phase separation method	161.9 mAh g^−1^, 200 cycles	4.7 V	-	0.8 mS cm^−1^	[49]
	LiFePO_4_	P(VDF-HFP)/(PEGMEMA)(MMA)(OVPOSS)-(PC)(ZnONP)	Li	High-speed stirring, UV radiation	145 mAh g^−1^, 100 cycles	0.5–4.2 V	-	1.4 mS cm^−1^	[83]
	LiFePO_4_	HP-A/BMIMBF_4_/PVDF/LiBF_4_	Li	Photopolymerization synthesis, solution casting	153.1 mAh g^−1^, maintained 98.1% after 100 cycles	4.3 V	-	From 1.2 mS cm^−1^ at 20 °C to 50 mS cm^−1^at 120 °C	[45]
	LiFePO_4_	IE-T	Li	Rapid sol–gel method	142 mAh g^−1^, 100 cycles	2.5–4.2 V	-	1.21 mS cm^−1^ at 30 °C	[30]
		IE-B			140 mAh g^−1^, 100 cycles			1.19 mS cm^−1^ at 30 °C	
	LiFePO_4_	PDEIm/P(VDF-HFP	Li	Simple and scalable phase inversion method	138.4, mAh g^−1^, 200 cycles	4.2 V	-	1.78 mS cm^−1^ at 25 °C	[84]
	LiFePO_4_	Li-TFSI/TEOS/(BMP)(TFSI)	Li	Sol–gel method	140 mAh g^−1^, 30 cycles	4.3 V	-	0. 34 mS cm^−1^	[32]
	LiFePO_4_	P(VDF-HFP)-NMP/Pyr_13_TFSI/LiTFSI	Li	NIPS	161,9 mAh g^−1^, 200 cycles	4.7 V	-	0.8 mS cm^−1^ at 25 °C	[49]
	LiCoO_2_	TMPTMA/(LiTFSI)(TBMA-TFSI-DMC)	Li	In situ thermal polymerization	149 mAh g^−1^ after 100 cycles	5.3 V	-	6.15 mS cm^−1^ at 25°C	[37]
	LiFePO_4_	P(VDF-HFP)/LiTFSI/PMPyrrTFSI	Li	Slurry-casting method	148.1 mAh g^−1^ after 100 cycles	Up to 5.6 V	-	6.93 mS cm^−1^ at 25°C	[50,51,52]
Li-metal battery	LiCoO_2_	P(VDF-HFP)/SiO_2_-(butanon)(NMP)- (EMIMTFSI/EC/PC)	Li	Electrodeposition	120 mAh g^−1^, 100 cycles	5 V	-	1.11 mS cm^−1^ at 25 °C	[88]
	LiFePO_4_	LiTFSI-EMIMTFSI-P(VDF-HFP)	Li-S	Melt diffusion method	867 mAh g^−1^, 200 cycles	4.5 V	-	0.88 mS cm^−1^ at room temperature	[48]
	Li_3_V_2_(PO_4_)_3_	PIL-GF-[EtO(CH_2_)_2_MMI]TFSI	Li	Electrodeposition	46 mAh g^−1^, 1000 cycles	3–4.3 V	-	0.56 mS cm^−1^ at room temperature	[89]
Li-polymer battery	Li_2_CuO_2_-LNMC	P(VDF-HFP)/PYR_13_FSI/LiTFSI	Li	Evaporation in dry box	90 mAh g^−1^, 100 cycles	4.3 V	-	1.6 mS cm^−1^ at 30 °C	[53]
Li–oxygen battery	LiTFSI in EMIMTFSI	PMMA/(MPSP)(EMIM-TFSI)	Li	Thermal curing	90 mAh g^−1^, 20 cycles	1 V	-	0.54 mS cm^−1^	[87]
Cu electrode	-	(TetraPEG) (LiTFSA/P_2225_/TFSA	-	Salting-in	-	Up to 4.2 V	-	-	[85]

**Table 3:** Electrochemical performances of various IL-based gels in different SCs.

SCs	IL-Based Gel	Preparation Process	Electrodes Used	Lifecycles and Capacity Retention	Stability Window	Specific Capacitance	Power and Energy Density	Ionic Conductivity	Refs.
EDLCs	EMIMTFSI/TMOS/DMDMS	Sol–gel method	AC	25,000 at 100 °C and 92%	0–3 V	177 mF cm 2 at 25 °C	-	4 mS cm^−1^ at 20 °C and 26 mS cm^−1^ at 100 °C	[29]
	BMIMPF_6_/DVIMBr/HEMA	Thermal polymerization	CNTs	-	3 V	1.5–3 mF cm^−2^	-	0.3 to 0.07 mS cm^−1^	[105]
	EMIMBF_4_/P(VDF-HFP)	Solution casting	rGO	12,000 cycles and 90%	-	242 F g^−1^ at 5 mV s^−1^	33 kW kg^−1^ and 92 Wh kg^−1^	140 mS cm^−1^	[121]
	BMIMTFSI/PVA/CH_3_COONH_4_	Solution casting	CNTs		3.3 V	3.36 F g^−1^	11.37 kW kg^−1^ and 0.17 W h kg^−1^	2.37 ± 0.02 mS cm^−1^ at 120 °C	[123]
	BMIMBF_4_/PVDF-HFP/SN/	Solution casting	AC	10,000 cycles and ~ 80%	−2.9–2.5 V	176 F g^−1^ at 0.18 A g^−1^ and 138 F g^−1^ at 8 A g^−1^	-	0.5 mS cm^−1^ at −30 °C to 15 mS cm^−1^ at 80 °C	[124]
LICs	P(VDF-HFP)/EMIMBF_4_/LiTFSI	Solution casting	NiO/rGO as the anode and AC as thecathode	4000 cycles and 83%	0–4 V	56.7 F g^−1^	0.8685 kW kg^−1^ and 49 Wh kg^−1^	-	[104]
	Glycidyl-POSS in 1 M LiTFSI/BMPTFSI	Thermal polymerization	AC	100 cycles and 81%	5 V vs. Li/Li^+^	-	-	0.701 mS cm^−1^ at 30 °C	[109]
	EMIMTFSI/LiTFSI/P(VDF-HFP)	Solution casting	AC	10,000 cycles and 93.7%	0–4 V	68.3 F g^−1^	2.47 kW kg^−1^ and 147.2 Wh kg^−1^ at 60 °C	-	[54]
	(PEO)-co-methacryloxypropyl) silsesquioxane/BMPTFSI/LiTFSI	Thermal polymerization	AC	50 cycles and 97%	-	-	-	0.62 mS cm^−1^ at 30 °C	[125]
Solid-state SCs	PVDF-HFP/BMIMI/CNTs	Solution casting	AC	10,000 cycles and 80.1%	-	15.7 F g^−1^	0.4599 kW kg^−1^ and 50.1 Wh kg^−1^	17.6 mS cm^−1^	[59,60,61]
	polyoxyethylene/nitrile butadiene rubber/EMIMTFSI	In situ polymerization	Graphene	10,000 cycles and 93.7%	0–2.5 V	208 F g^−1^ at a current density of 1 A g^−1^	5.87 kW h kg^−1^ and 181 W h kg^−1^	2.4 mS cm^−1^ at room temperature	[126]
	polyacrylamide/1-vinyl-3-methylimidazolium bis(trifluoromethylsulfonyl)imide	Polymerization	Poly(3,4-ethylenedioxythiophene/carbon cloth	3000 cycles and 93%	-	157.8 F g^−1^	14.22 Wh kg^−1^	23 mS cm^−1^ at 90 °C	[1]
Solid-state sodium ion SCs	P(VDF-HFP)/EMIMBF_4_/NaTFSI	Solution casting	MoS_2_/CNTs as working electrode and sodium foil used as a counter electrode	8000 cycles and 81%	0–3.8 V	-	2.0909 kW kg^−1^ and 80 W h kg^−1^ at 70 °C and	0.67 mS cm^−1^ at 20 °C	[57,58]
Self-charging SCs	P(VDF-HFP)/TEABF_4_	Electrospinning	MoSe_2_ electrodes	2500 cycles and 91%	0–2 V	18.93 mF cm^−2^	268.91 μW cm^−2^ and 37.90 mJ cm^−2^		[118]
Flexible SCs	P(VDF-HFP)/EMIMTFSI	Sol–gel method	Carbon electrode	10,000 cycles and 99.95%	0–2.5 V	118 to 115 F g^−1^	6.25 kW kg^−1^ and 21.9 W h kg^−1^	8.6 mS cm^−1^	[35]
	PHEMA-co-PEGDMA/EMIMBF_4_	UV-polymerization	AC	3500 cycles and 91%	0–3 V	193.33 F g^−1^ at 5 mV s^−1^	1.23 kW kg^−1^ and 49.55 Wh kg^−1^	12.27 mS cm^−1^ at room temperature	[115]
	P(VDF-HFP)/EMIMBF_4_	Solution casting	MnO_2_/CNTs	10,000 cycles and 88.5%	3 V	8.6 F cm^−3^	1804 mWcm^−3^ and 4.2 mWh cm^−3^	-	[127]
	PVA/boric acid/EMIMCl	Solution casting	AC	3000 cycles and 98%	0–1 V	90 F g^−1^	0.9 kW kg–1 and 12.36 Wh kg–1	2.43 mS cm^−1^	[128]
MSCs	Fumed SiO_2_/EMIMTFSI	Sol–gel method	Porous carbon	10,000 cycles and 94%	2 V	-	26 W cm^−3^ and mW h cm^−3^	-	[110]
	PVDF/EMIMTFSI	Sol–gel method	3D MnO_2_ electrodes	30,000 cycles and 96.2%	-	36 mF cm^−2^ at 20 mV s^−1^	3.8 mW cm^−2^ and 4.4 μWh cm^−2^	-	[121]
	P(VDF-HFP)/EMIMBF_4_	Solution casting	Graphite as working electrode and Pt foil as counter electrode	5000 cycles and 93%	3.5 V	17.4 mF cm^−2^	59 mWh cm^−3^	25 mS cm^−1^	[122]
	PEGDA/EMIMTFSI	UV-polymerization	Multi-walled carbon nanotube (MWCNT)	30,000 cycles and ~80%	0–2 V	5.3 F cm^−3^ 10 mV s^−1^	21.0 W cm^−3^ and 0.17 mWh cm^−3^	9.4 mS cm^−1^	[129]
	PEO/BMIMTFSI/LiTFSI	Solution casting	CNT microelectrodes	5000 cycles and 94.4%	0–3 V	21 F g^−1^	750 μW cm^−2^ and 47.88 μWh cm^−2^	4.07 mS cm^−1^	[130]
	Microporous carbon/Fe_2_O_3_/EMIMBF_4_	3D printing	Microporous carbon/Fe_2_O_3_	10,000 cycles and 93.2%	-	377 F g^−1^	-	-	[131]

## Data Availability

Not applicable.
